# Timing of Fascial Plane Blocks in Bariatric Surgery: Pre-Incision Administration Is Associated with Lower Early Pain and Opioid Consumption

**DOI:** 10.3390/jcm15187204

**Published:** 2026-09-16

**Authors:** Alper Tunga Doğan, Sami Kaan Coşarcan, Yunus Yavuz, Ömür Erçelen

**Affiliations:** 1 Department of Anesthesiology and Pain Clinic, VKV American Hospital, 34365 Istanbul, Turkey; 2 Department of Anesthesiology and Reanimation, Koç University School of Medicine, 34010 Istanbul, Turkey; 3 Department of General Surgery—Bariatric and Metabolic Surgery Unit, VKV American Hospital, 34365 Istanbul, Turkey; 4 Department of General Surgery, Koç University School of Medicine, 34010 Istanbul, Turkey

**Keywords:** bariatric surgery, fascial plane block, block timing, postoperative pain, quality of recovery, QoR-15, opioid sparing, external oblique intercostal block

## Abstract

**Background:** Fascial plane blocks reduce opioid consumption after bariatric surgery, but the optimal timing of administration remains unclear. We compared pre-incision versus end-of-surgery block timing. **Methods:** This was a retrospective observational cohort study of consecutive patients undergoing laparoscopic sleeve gastrectomy at a single centre. All patients received a left external oblique intercostal block plus a bilateral rectus sheath block with 0.25% bupivacaine, performed by the same two experienced anaesthesiologists either before incision or at the end of surgery. Ward staff who routinely recorded postoperative pain scores did not have access to the anaesthesia record and were therefore unaware of block timing. The primary outcome was the numeric rating scale (NRS) pain score at post-anaesthesia care unit (PACU) admission. Repeated pain scores were analysed using generalized estimating equations (GEE) with an exchangeable correlation structure and robust standard errors, adjusted for BMI, ASA status and anaesthesia duration, and group  ×  time interaction was tested. Secondary outcomes included 24 h intravenous morphine milligram equivalents (IV-MME) and the 24 h Quality of Recovery-15 (QoR-15) score, analysed with adjustment for baseline. **Results:** Fifty-four patients were analysed (pre-incision *n* = 28, post-surgery *n* = 26). Median NRS at PACU admission was lower in the pre-incision group (4.5 [IQR 2.0–8.0] vs. 7.0 [IQR 7.0–8.0]; *p* = 0.008). In the adjusted GEE model, pre-incision timing was associated with a 1.53-point lower NRS across the postoperative period (95% CI −2.15 to −0.91; *p* < 0.001). The between-group difference attenuated over time, from −2.42 points (95% CI −3.72 to −1.12) at PACU admission to −0.81 points (95% CI −1.30 to −0.33) at 24 h, although the group × time interaction did not reach significance (*p* = 0.075). PACU rescue fentanyl (median 62 [IQR 25–106] vs. 150 [IQR 100–194] µg; *p* < 0.001) and 24 h PCA tramadol (median 120 [IQR 100–150] vs. 172 [IQR 141–199] mg; *p* < 0.001) were both lower in the pre-incision group; mean consumption was 44% and 26% lower, respectively, corresponding to a 35% lower mean total IV-MME. No statistically significant difference in quality of recovery was detected, and the estimate was imprecise; the baseline-adjusted 24 h QoR-15 difference was 5.3 points (95% CI −7.0 to 17.6; *p* = 0.391). An exploratory subdomain analysis suggested better physical comfort with pre-incision timing, but this did not survive correction for multiple comparisons. **Conclusions:** In this retrospective cohort, pre-incision administration of fascial plane blocks was associated with lower early postoperative pain and lower recorded opioid consumption than end-of-surgery administration, with no statistically significant difference in overall quality of recovery, although that estimate was imprecise. Because block timing was determined by operating-room logistics rather than by randomisation, and intraoperative opioid exposure could not be quantified, these associations require confirmation in a prospective randomised trial before they can guide practice.

## 1. Introduction

Bariatric surgery is the most effective treatment for clinically severe obesity and its associated comorbidities [1,2]. Despite the use of minimally invasive laparoscopic techniques, patients often report moderate-to-severe pain in the early postoperative period [3]. In this population, the high prevalence of obstructive sleep apnoea and the risk of opioid-induced respiratory depression mandate strict limitation of opioid use [4,5]; multimodal opioid-sparing analgesia is therefore central to enhanced recovery programs in bariatric surgery [6,7]. In sleeve gastrectomy specifically, combining opioid-free anaesthesia with loco-regional techniques within an enhanced recovery pathway has been shown to improve patient-reported quality of recovery [8].

Fascial plane blocks are practical tools within this multimodal approach, including the established transversus abdominis plane block [9,10] and the more recently described external oblique intercostal (EOI) block [11]; comparative trials and meta-analyses continue to refine the choice among these techniques [12,13]. In a previous study from our centre, we found that fascial plane blocks substantially reduced postoperative opioid consumption in 113 patients undergoing laparoscopic bariatric surgery [14]. Two techniques demonstrated superior efficacy: the combination of transversus abdominis plane and rectus sheath block, and the EOI block. The EOI block offers a practical advantage in this population; its target plane lies superficial to the bulk of truncal subcutaneous fat at the lateral chest wall, remaining accessible under ultrasound even in patients with severe obesity [14,15]. Since 2024, several randomised controlled trials have evaluated the EOI block in laparoscopic sleeve gastrectomy, against a control group [16], against port-site infiltration [17], against an alternative fascial plane technique [18], and in a further placebo-controlled trial [19], and a recent meta-analysis supports its opioid-sparing effect [20]. However, all of these trials administered the block at a single predetermined time point, and none has compared the timing of block administration. Whether placing an established block before incision versus at the end of surgery affects early recovery remains unaddressed by prospective evidence. A recent narrative review of nerve block techniques in bariatric surgery likewise proposes that pre-incisional administration of the transversus abdominis plane block may be more effective than post-incisional placement, through a pre-emptive analgesic mechanism that limits the establishment of altered sensory processing in response to surgical stimulus, while noting that this proposition has not been tested directly [21].

Following integration of fascial plane blocks into routine bariatric anaesthesia at our institution, a recurrent pattern emerged; patients who received blocks at the end of surgery consistently reported significant discomfort in the first 30–60 min after emergence, despite adequate ward-level pain control. This clinical observation prompted the hypothesis that blocks performed at wound closure might not have reached their full analgesic effect by the time of emergence. Pre-incision placement was therefore considered preferable when operating-room workflow permitted; in routine practice, however, the actual timing continued to depend primarily on the availability of the regional anaesthesia team.

Quality of Recovery-15 (QoR-15) is a validated, patient-reported outcome measure covering five domains: physical comfort, emotional state, physical independence, psychological support, and pain [22,23]. Its reliability and responsiveness have been demonstrated across diverse surgical populations [24,25], with a minimal clinically important difference of 6.0–8.0 points on a 0–150 scale [26]. Unlike unidimensional pain scores, the QoR-15 captures aspects of recovery directly relevant to enhanced recovery programs [27].

The aim of this study was to examine whether the timing of fascial plane block administration, pre-incision versus end-of-surgery, was associated with differences in early postoperative pain, opioid consumption and quality of recovery in patients undergoing laparoscopic sleeve gastrectomy.

## 2. Materials and Methods

### 2.1. Study Design and Ethical Approval

This retrospective cohort study was conducted at VKV American Hospital, Istanbul, Turkey, with approval from the Koç University Clinical Research Ethics Committee (2025.237.IRB2.105). The study was performed in accordance with the 1964 Helsinki Declaration. Ethical approval was granted on 27 May 2025, before any retrospective data extraction or analysis was undertaken. Individual informed consent was waived given the retrospective design.

### 2.2. Patient Selection

**Figure 1:**
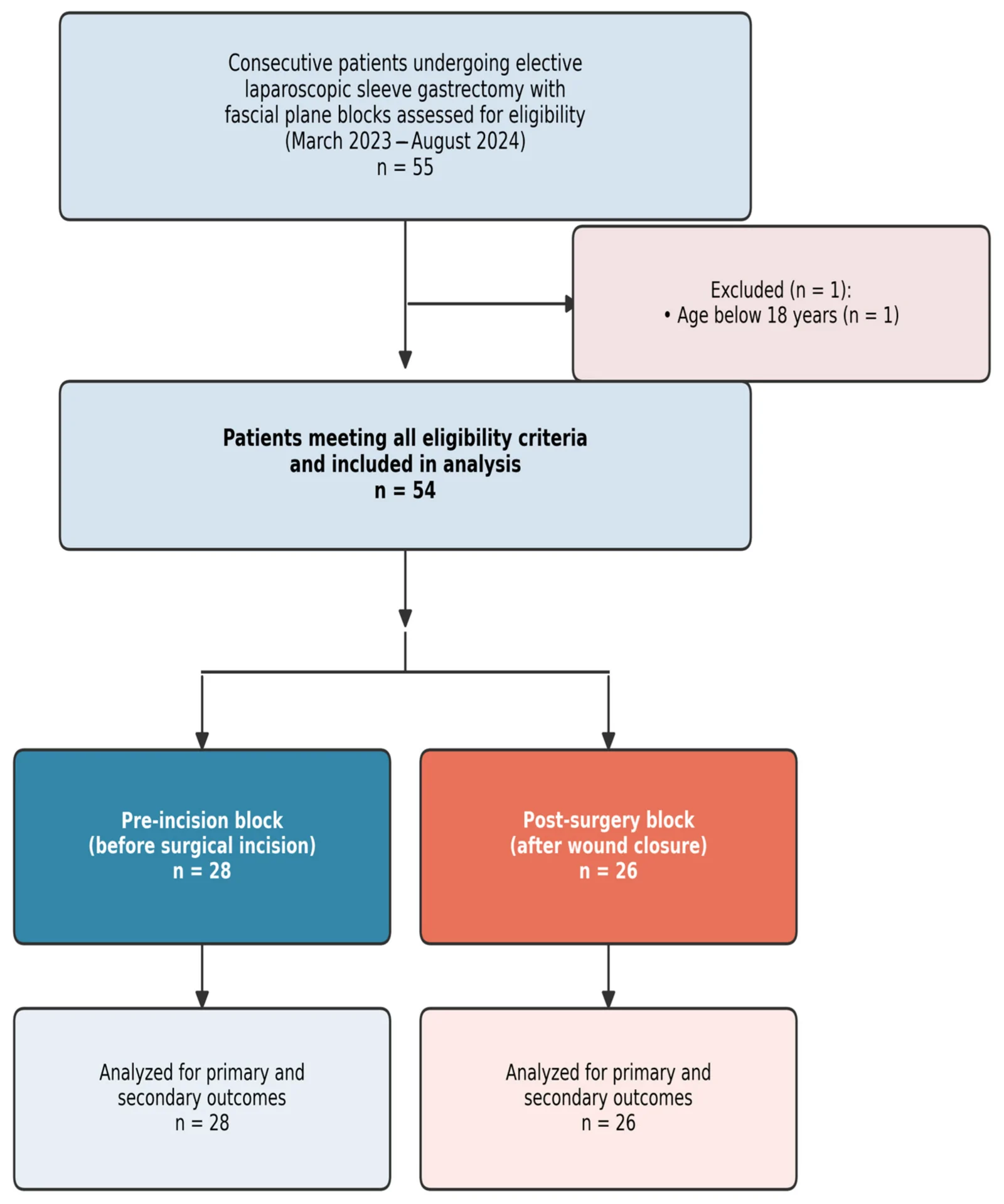
Study flow diagram. Consecutive adult patients undergoing elective laparoscopic sleeve gastrectomy with combined external oblique intercostal and bilateral rectus sheath blocks between March 2023 and August 2024 were assessed for eligibility (*n* = 55). One patient was excluded because of age below 18 years, leaving 54 patients for analysis. Patients were classified according to the documented timing of block administration: before surgical incision (pre-incision group, *n* = 28) or after wound closure while still under general anaesthesia (post-surgery group, *n* = 26). Block timing was primarily determined by the availability of the regional anaesthesia team on the day of surgery rather than by a predefined clinical criterion. All 54 patients had complete pain and Quality of Recovery-15 data and were included in the analysis of primary and secondary outcomes; no patients were lost to follow-up.

We reviewed the records of all consecutive adult patients (≥18 years) who underwent elective laparoscopic sleeve gastrectomy between March 2023 and August 2024. All procedures were laparoscopic sleeve gastrectomy performed by the same surgical team using a standardised five-port technique; no robotic, open, or other bariatric procedures (e.g., gastric bypass) were included, ensuring a homogeneous surgical cohort. Patients with ASA physical status II–III and complete perioperative documentation were eligible. We excluded patients with chronic pain or long-term opioid use, conversions to open surgery, revisional bariatric procedures, and those with incomplete pain or QoR-15 records. Records with incomplete pain or QoR-15 documentation were excluded at the eligibility stage rather than after outcome assessment, and all patients entering the analysis had complete data for every reported outcome; no imputation was therefore required. During the record review, one patient found to be below 18 years of age was excluded, leaving 54 patients for analysis (Figure 1).

### 2.3. Group Allocation

During the study period, all patients undergoing laparoscopic bariatric surgery at our institution routinely received fascial plane blocks as part of a standardised multimodal analgesia pathway. No institutional protocol mandated when the block should be performed, and the decision was not based on a predefined patient-specific clinical criterion. Timing was not informed by body mass index, ASA physical status, comorbidity burden, anticipated case complexity, or any other patient characteristic. Timing was instead governed by the availability of the regional anaesthesia team on the day of surgery. All blocks were performed by the same two anaesthesiologists. When one of them was free at the time of anaesthetic induction, the block was placed before surgical incision; when both were committed to a concurrent case in another operating room, or when a preceding operation over-ran and the list was compressed, the block was placed after wound closure with the patient still under general anaesthesia. Both anaesthesiologists administered blocks at both timings; so, operator experience did not differ systematically between the groups. For this retrospective analysis, patients were classified into two groups according to the documented timing of block administration. Ward nursing staff who collected postoperative pain scores and QoR-15 data did not have access to anaesthetic records and were unaware of block timing.

### 2.4. Anaesthetic and Analgesic Protocol

Anaesthetic and analgesic management was extracted from institutional records. Procedures followed routine institutional practice: intravenous induction with propofol and fentanyl, maintenance with desflurane or sevoflurane, and remifentanil titrated to surgical stimulation. Neuromuscular blockade was with rocuronium and reversed with sugammadex.

All patients received intravenous ibuprofen 800 mg, paracetamol 1 g, and tramadol 100 mg before emergence, regardless of block timing. This regimen reflects the institutional protocol established in our previous study [14]; consistent application was confirmed through retrospective review of anaesthetic records.

### 2.5. Fascial Plane Block Technique

**Figure 2:**
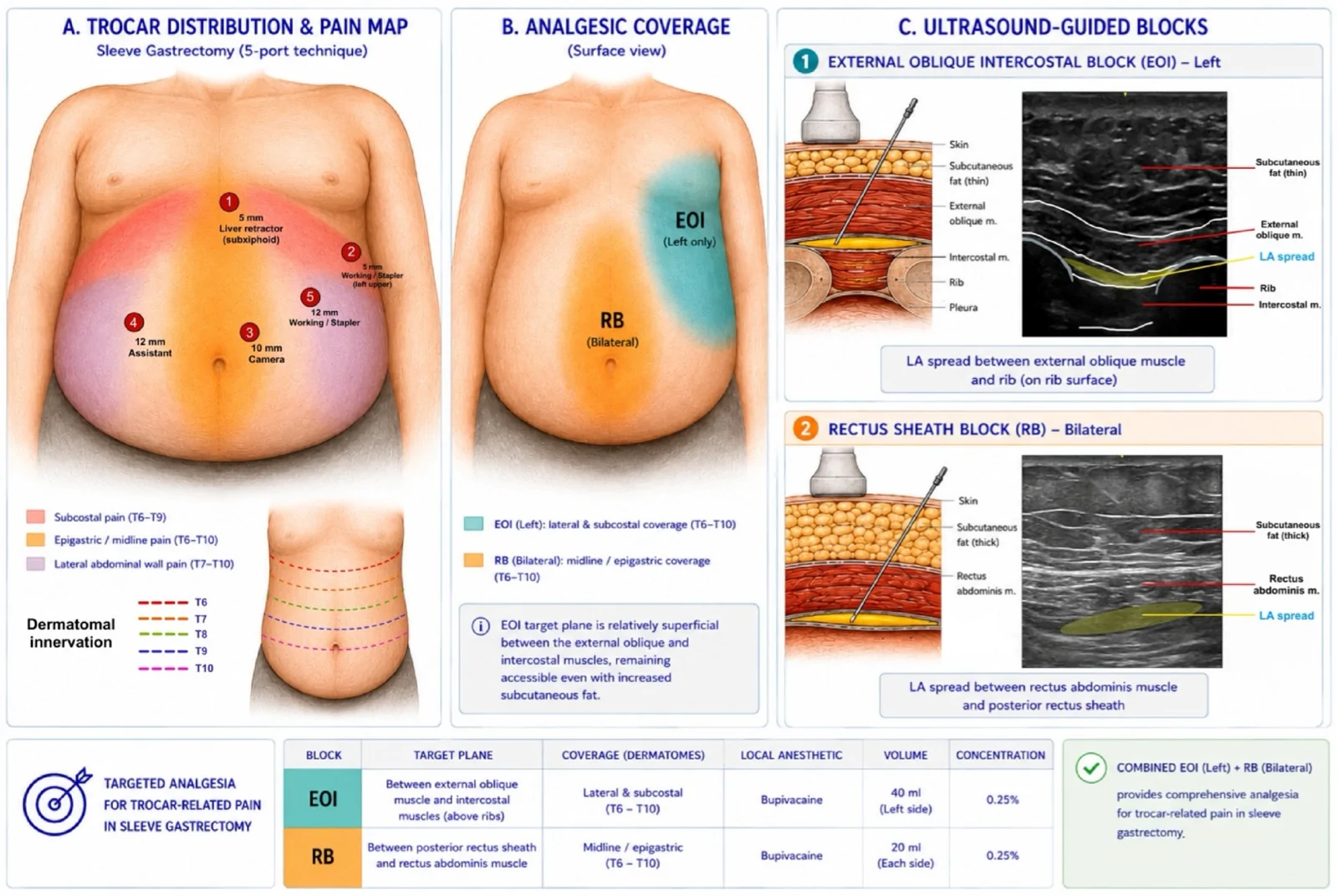
Combined external oblique intercostal (EOI) and rectus sheath block technique for laparoscopic sleeve gastrectomy. (**A**) Trocar distribution and corresponding pain map for the five-port technique used in this study; one 5 mm subxiphoid liver retractor, one 5 mm left upper working/stapler port, one 12 mm working/stapler port, one 10 mm camera port, and one 12 mm assistant port. Color overlay indicates the typical somatic pain distribution from trocar sites (T6–T10 dermatomes). (**B**) Analgesic coverage of the combined block. The left EOI block targets the lateral and subcostal abdominal wall (T6–T10); bilateral rectus sheath blocks target the midline/epigastric region (T6–T10). The EOI target plane lies superficial to the bulk of truncal subcutaneous fat and remains accessible under ultrasound guidance in patients with obesity. (**C**) Ultrasound-guided block technique. (C1) EOI block: in-plane needle approach between the external oblique muscle and the intercostal muscle/rib surface; (C2) Rectus sheath block: in-plane approach between the rectus abdominis muscle and the posterior rectus sheath. Local anesthetic doses: EOI 40 mL of 0.25% bupivacaine (left); rectus sheath 20 mL of 0.25% bupivacaine on each side. LA, local anesthetic. Figure design by Deniz Akbay, MD, with the assistance of ChatGPT 5.5 (OpenAI).

Two experienced anaesthesiologists performed all blocks under real-time ultrasound guidance (GE LOGIQ P9 R3; linear probe, 4–12 MHz) with a 22 G, 80 mm insulated needle (Braun Sonoplex, Melsungen, Germany). The protocol [14] consisted of a left-sided EOI block with 40 mL of 0.25% bupivacaine combined with bilateral rectus sheath blocks at the umbilical level (20 mL of 0.25% bupivacaine per side). Total bupivacaine volume was 80 mL (200 mg). The operating surgeon additionally infiltrated 3 to 5 mL of 0.25% bupivacaine at each of the five trocar insertion sites (one 5-mm subxiphoid liver retractor port plus four working trocars of 5–12 mm in the standard sleeve gastrectomy configuration) before placement. Trocar-site infiltration therefore contributed a further 37.5 to 62.5 mg. Combining both sources, the mean total bupivacaine dose was 2.28 mg/kg (range 1.14 to 3.65 mg/kg), calculated on total body weight. The block component was administered by the anaesthesiologist at induction or at the end of surgery, and the trocar-site infiltration by the surgeon at the time of port placement. The implications of this dosing for local anaesthetic safety are considered in the Discussion. The external oblique intercostal block was performed on the left side only. This reflected the trocar distribution of our standardised five-port technique, in which the stapling ports and the majority of the lateral subcostal incisions lie on the left, while midline and epigastric port pain is covered by the bilateral rectus sheath blocks; most published external oblique intercostal trials in sleeve gastrectomy have instead used bilateral blocks, and this difference should be considered when comparing techniques across studies. In the pre-incision group, blocks were placed after anaesthetic induction but before skin incision; in the post-surgery group, after the procedure was completed, while the patient remained under general anaesthesia (Figure 2).

### 2.6. Postoperative Pain Management

In the PACU, breakthrough pain was treated with intravenous fentanyl, 25 µg for NRS < 7 or 50 µg for NRS ≥ 7. On the ward, all patients received intravenous paracetamol 1 g every 6 h until discharge plus a tramadol patient-controlled analgesia (PCA) device programmed for demand boluses only, with no background infusion (10 mg bolus, 15-min lockout). No additional rescue analgesics were administered. Cumulative PCA tramadol consumption was recorded by the acute pain nursing team during routine ward rounds and transcribed into the patient chart; these chart entries were the source of the tramadol data reported here.

### 2.7. Outcome Measures

The primary outcome was the NRS pain score at rest at PACU admission (0 min). Secondary outcomes included NRS at subsequent time points (15, 30, 45, 60 min, 6 and 24 h), PACU fentanyl consumption, total PCA tramadol consumption, total 24 h intravenous morphine milligram equivalents (IV-MME), and the 24 h QoR-15 total score. The change in QoR-15 from baseline to 24 h (Δ QoR-15) was additionally calculated for the exploratory correlation analyses. IV-MME was calculated using equianalgesic dose ratios appropriate for the acute care setting (fentanyl 100 µg IV = morphine 10 mg IV; tramadol 100 mg IV = morphine 10 mg IV); these ratios reflect single-dose equipotency in the postoperative period rather than long-term steady-state equivalence [28].

QoR-15 was completed by patients at the preoperative outpatient visit (baseline) and at 24 h after surgery, using the validated Turkish version (QoR-15T) [29,30]. Exploratory outcomes included QoR-15 subdomain analysis across the five dimensions.

### 2.8. Statistical Analysis

Continuous variables were tested for normality with the Shapiro–Wilk test and reported as mean ± SD; between-group comparisons used the independent samples *t*-test or Mann–Whitney U test as appropriate. Categorical variables were compared using Fisher’s exact test. Effect sizes are reported as the Hodges–Lehmann median difference with its distribution-free 95% confidence interval and the rank-biserial correlation for non-parametric comparisons; Cohen’s d is additionally reported for the primary outcome to allow comparison with earlier studies. To account for the correlation between repeated NRS measurements within patients, repeated pain scores across all seven postoperative time points were analysed using generalized estimating equations (GEEs). The models specified a Gaussian distribution with an identity link, with time entered as a categorical factor (PACU admission as the reference level) and robust (sandwich) standard errors. An exchangeable working correlation structure was used; the estimated within-patient correlation was 0.32, and the quasi-likelihood information criterion was closely similar for independent, exchangeable and first-order autoregressive structures (QIC 388.4, 388.4 and 388.1, respectively); so, the exchangeable structure was retained as the pragmatic choice for unequally spaced measurements. An unadjusted model (group and time) and an adjusted model were fitted. The adjusted model included BMI, ASA physical status (entered as a binary indicator, III versus II) and anaesthesia duration. These three covariates were selected a priori on clinical grounds, before the analyses were run, because each is plausibly related both to perioperative management and to postoperative pain in bariatric surgery, and because the sample size of 54 patients precluded extensive multivariable adjustment. A separate model including a group  ×  time interaction was fitted to examine whether the between-group difference varied across the postoperative period; the interaction was assessed with a multi-degree-of-freedom Wald test, and model-based between-group differences with 95% confidence intervals were derived at each time point. Results are reported as regression coefficients with 95% confidence intervals. The primary analysis of the QoR-15 compared the 24 h total score between groups using analysis of covariance, with the baseline QoR-15 score entered as a covariate; baseline-adjusted analyses were performed in the same way for Part A, Part B and the five subdomains, and are reported as adjusted between-group differences with 95% confidence intervals. Clinical relevance was interpreted against the published MCID of 6.0–8.0 points for the total scale [26,31]. Subdomain comparisons were considered exploratory; the Benjamini–Hochberg procedure was applied across the five subdomains to control the false discovery rate at 5%, with both raw and adjusted *p*-values reported. Correlations were assessed with Spearman’s ρ. All tests were two-sided with α = 0.05.

### 2.9. Use of Generative Artificial Intelligence

**Figure 3:**
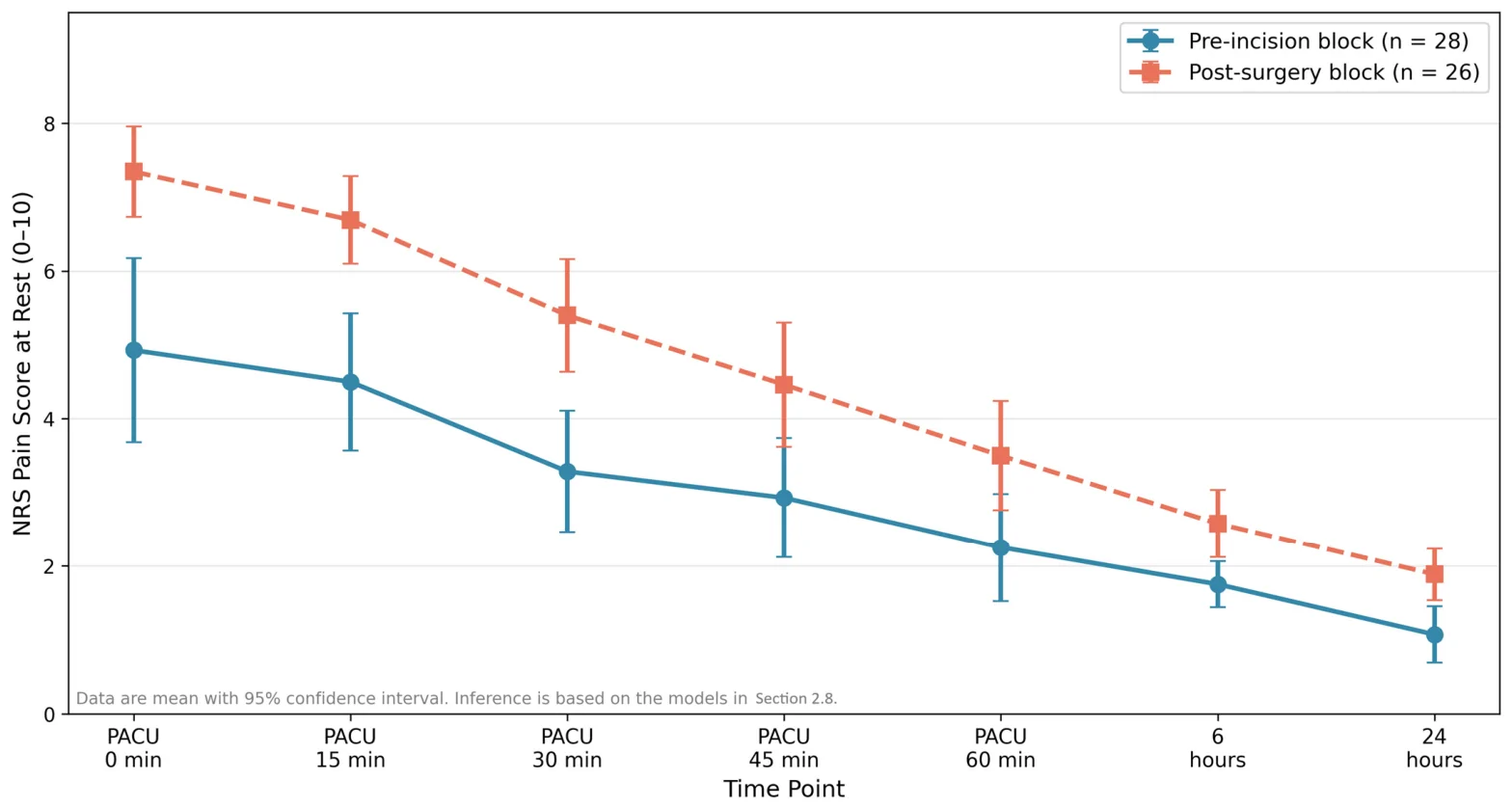
NRS pain scores at rest over time. Data are mean with 95% confidence interval. Measurements begin at PACU admission; no preoperative pain score was collected. Inference for the repeated measurements is based on the models presented in Section 2.8.

**Figure 4:**
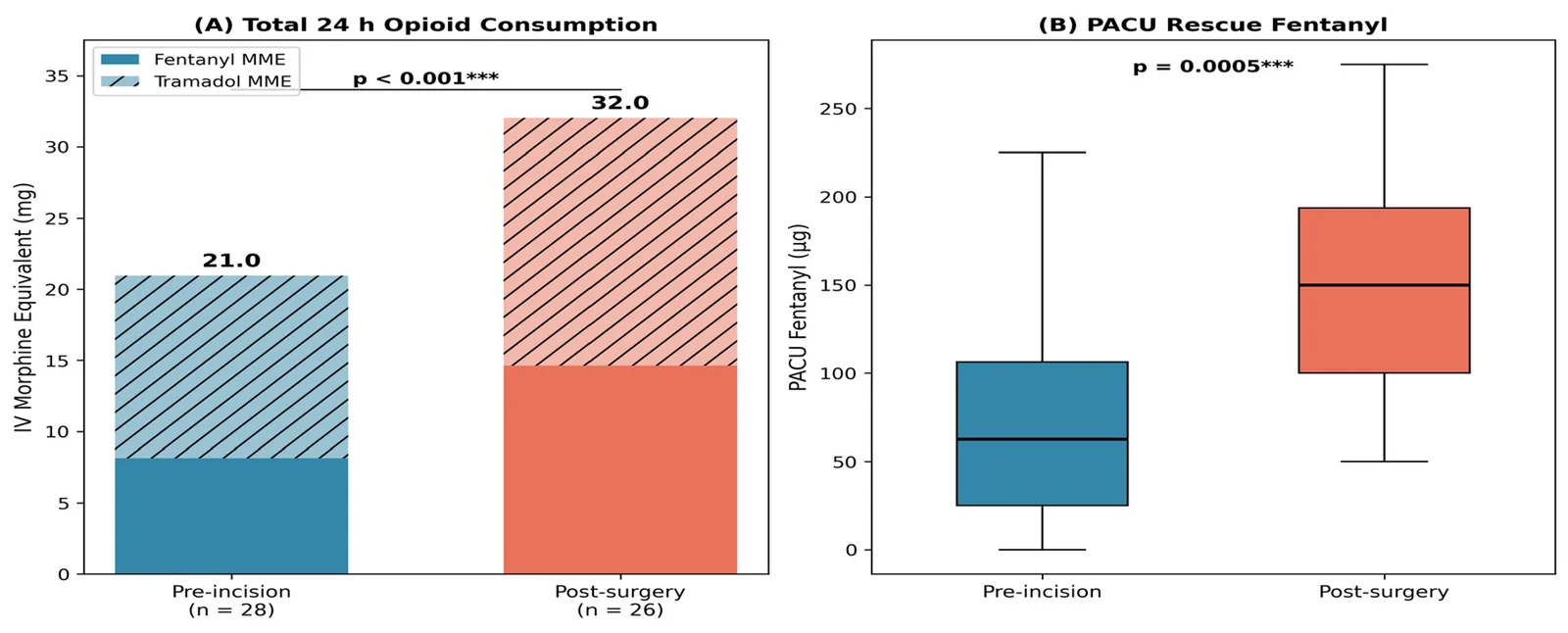
Opioid consumption. (**A**) Total 24 h intravenous morphine milligram equivalents. (**B**) PACU rescue fentanyl. *** *p* < 0.001. In panel (**A**), blue and orange bars denote the pre-incision and post-surgery groups, respectively; solid and hatched segments denote the fentanyl and tramadol contributions to total IV-MME.

**Figure 5:**
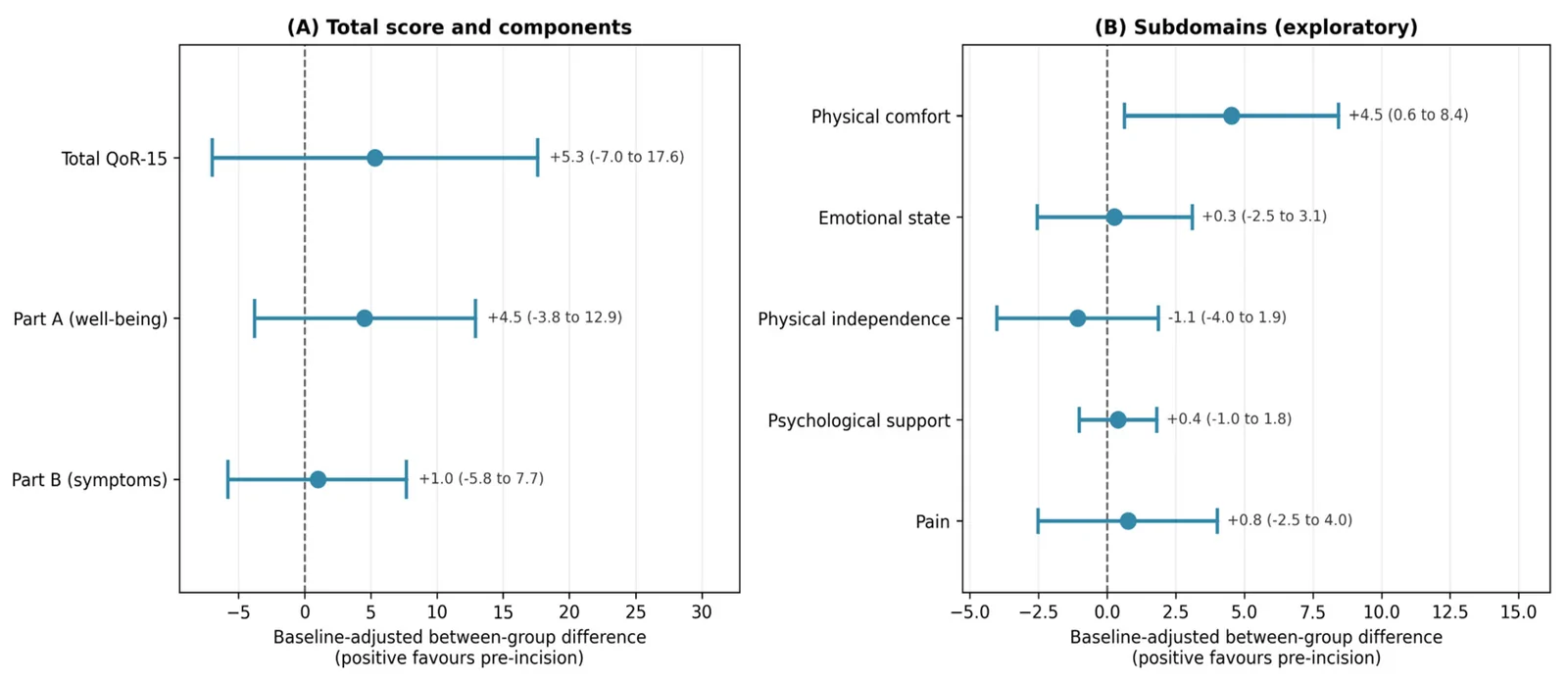
Baseline-adjusted between-group differences in Quality of Recovery-15 scores. (**A**) Total score, Part A and Part B. (**B**) The five subdomains, analysed as exploratory outcomes. Points are adjusted differences from analysis of covariance with the corresponding baseline score as covariate; bars are 95% confidence intervals. Positive values favour pre-incision timing. The published minimal clinically important difference of 6.0 to 8.0 points applies to the total scale only. Note the different horizontal scales in the two panels. No subdomain remained statistically significant after Benjamini–Hochberg correction across the five exploratory comparisons.

**Figure 6:**
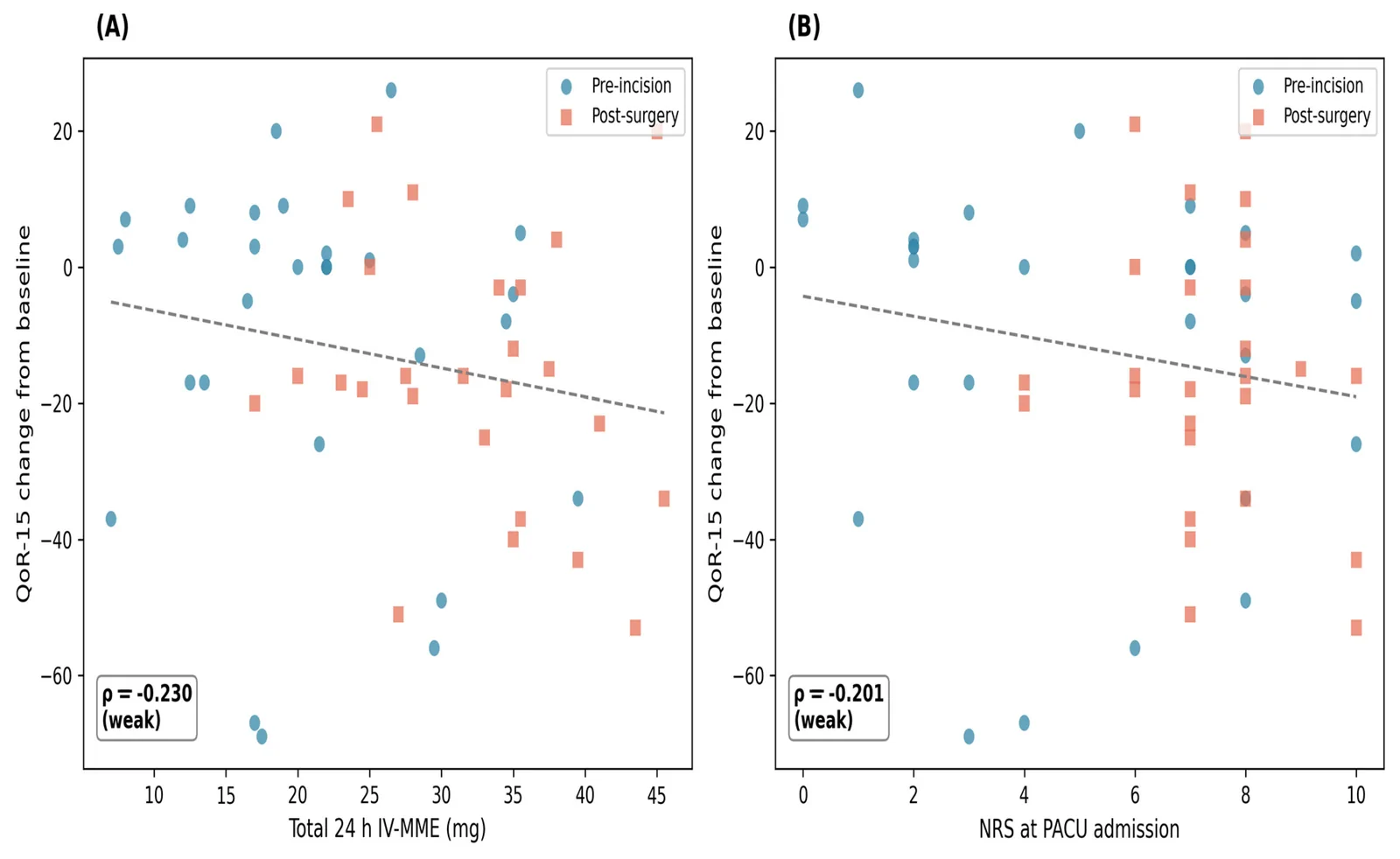
Correlation between analgesic outcomes and quality of recovery. (**A**) Total 24 h IV-MME versus change in QoR-15 from baseline. (**B**) NRS at PACU admission versus change in QoR-15 from baseline. Both correlations are exploratory and were weak and non-significant. Dashed lines indicate simple linear trend lines for visualization only; correlation inference is based on Spearman’s ρ.

Generative artificial intelligence tools were used during the preparation of this manuscript in two ways. First, for English-language editing, technical formatting of the text, tables and reference list, and checking of numerical consistency between the text and the tables. Second, for assistance with the preparation of the graphical elements of the explanatory schematic shown in Figure 2, which was designed by Deniz Akbay, MD; the anatomical content, the block descriptions and the dosing information shown in that figure were specified and verified by the authors. The tools were not used to generate, analyse or interpret the study data, to produce statistical results, to draft the scientific content or conclusions or to select or summarise the cited literature. All statistical analyses were performed by the authors, and all figures presenting study data (Figure 3, Figure 4, Figure 5 and Figure 6) were generated by the authors directly from the primary dataset. Every passage and image produced with AI assistance was reviewed, verified and edited by the authors, who take full responsibility for the entire content of this manuscript.

## 3. Results

### 3.1. Patient Characteristics

Of 55 consecutive eligible patients, one was excluded for age below 18 years, leaving 54 for analysis. Of these, 28 received blocks before incision and 26 at the end of surgery. The groups did not differ in age (39.5 ± 13.3 vs. 36.7 ± 13.9 years; *p* = 0.467), sex (male/female: 12/16 vs. 12/14; *p* = 1.000), BMI (42.0 ± 6.7 vs. 40.7 ± 5.6 kg/m^2^; *p* = 0.782), operative time (163 ± 38 vs. 171 ± 37 min; *p* = 0.456), or preoperative QoR-15 scores (124.4 ± 18.7 vs. 123.2 ± 17.7; *p* = 0.842). Documented comorbidity was also comparable: hypertension 12/28 (42.9%) versus 9/26 (34.6%; *p* = 0.586), diabetes 7/28 (25.0%) versus 7/26 (26.9%; *p* = 1.000), and any documented comorbidity 18/28 (64.3%) versus 14/26 (53.8%; *p* = 0.580). Obstructive sleep apnoea was documented in the medical record of 2/28 (7.1%) and 1/26 (3.8%) patients, respectively (*p* = 1.000); as no systematic screening was performed, this figure reflects previously diagnosed disease only and almost certainly underestimates the true prevalence in a bariatric population (Table 1). To assess whether one timing approach was applied systematically earlier in the study period (which could have introduced confounding through changes in surgical or anaesthetic experience over time), median surgical dates were compared (Mann–Whitney U, *p* = 0.409); a chi-square test of date categories by calendar year similarly showed no temporal difference (χ^2^ = 0.04; *p* = 0.838; Table 1). The two timings were also evenly distributed across calendar quarters (χ^2^ = 7.32; *p* = 0.292) and across days of the week (χ^2^ = 4.03; *p* = 0.545), and anaesthesia duration did not differ between groups (214 ± 35 vs. 202 ± 31 min; *p* = 0.371), arguing against systematic clustering of one timing on particular operating days or case types.

### 3.2. Primary Outcome: NRS at PACU Admission

The pre-incision group arrived in the PACU with lower pain scores than the post-surgery group (median NRS 4.5 [IQR 2.0–8.0] versus 7.0 [IQR 7.0–8.0]; Mann–Whitney U = 211.5; *p* = 0.008). The Hodges–Lehmann median difference was −2.5 points and the rank-biserial correlation was 0.42. Corresponding means were 4.9 ± 3.2 and 7.3 ± 1.5 (Cohen’s d = 0.95). Because the NRS distributions were skewed and bounded, non-parametric methods were used for inference; means are also given for comparability with earlier studies.

### 3.3. Secondary Pain Outcomes

Descriptive NRS values at each time point are shown in Table 2 and Figure 3. Inference was based on the repeated-measures model rather than on separate pointwise tests. In the unadjusted GEE model, the pre-incision group had a 1.59-point lower NRS on average across the postoperative period (95% CI −2.23 to −0.95; *p* < 0.001); after adjustment for BMI, ASA status and anaesthesia duration, the estimate was essentially unchanged (−1.53; 95% CI −2.15 to −0.91; *p* < 0.001). Complete model coefficients are given in Table 3. The covariates were included for confounding adjustment rather than for hypothesis testing, and their individual coefficients are reported in Table 3 without interpretation as independent predictors. Model-based between-group differences at each time point are presented in Table 4. The difference was largest at PACU admission (−2.42 points; 95% CI −3.72 to −1.12) and attenuated progressively to −0.81 points (95% CI −1.30 to −0.33) at 24 h. The group × time interaction did not reach statistical significance (Wald χ^2^ = 11.47; df = 6; *p* = 0.075), and this study was not powered to detect an interaction; the point estimates nonetheless describe a consistent monotonic attenuation of the difference over the first postoperative day. As a sensitivity analysis, pointwise Mann–Whitney comparisons at the seven time points remained significant after Holm correction (adjusted *p* = 0.003 to 0.023).

### 3.4. Opioid Consumption

The two opioids are reported separately as the directly observed outcomes. PACU rescue fentanyl was lower in the pre-incision group (median 62 µg [IQR 25–106] vs. 150 µg [IQR 100–194]; Hodges–Lehmann difference −75 µg, 95% CI −100 to −25; *p* < 0.001), a 44% lower mean dose (81 ± 67 vs. 146 ± 56 µg). PCA tramadol over 24 h was also lower (median 120 mg [IQR 100–150] vs. 172 mg [IQR 141–199]; Hodges–Lehmann difference −45 mg, 95% CI −60 to −20; *p* < 0.001), a 26% lower mean dose (128 ± 37 vs. 174 ± 45 mg). Expressed as a standardised summary measure, total 24-h IV-MME was 35% lower in the pre-incision group (21.0 ± 8.8 vs. 32.0 ± 7.7 mg; Hodges–Lehmann difference −11.0 mg, 95% CI −16.0 to −6.5; *p* < 0.001; Figure 4). The IV-MME value should be interpreted as a standardised summary rather than a precisely measured biological quantity, since equianalgesic conversion ratios, particularly for tramadol, carry appreciable uncertainty [28].

### 3.5. Quality of Recovery

Both groups started with similar QoR-15 scores. At 24 h, mean total scores were 113.5 ± 29.4 in the pre-incision group and 107.3 ± 21.9 in the post-surgery group. In the primary analysis of this outcome, an analysis of covariance of the 24 h score adjusted for the baseline score gave a between-group difference of 5.3 points in favour of pre-incision timing (95% CI −7.0 to 17.6; *p* = 0.391). The confidence interval spans zero and includes values both below and above the published minimal clinically important difference of 6.0 to 8.0 points, so that this study cannot exclude a clinically relevant effect in either direction; the result is best described as inconclusive rather than as evidence of no difference. Corresponding baseline-adjusted differences were 4.5 points for Part A (well-being; 95% CI −3.8 to 12.9; *p* = 0.282) and 1.0 point for Part B (symptoms; 95% CI −5.8 to 7.7; *p* = 0.778). Patient-reported nausea at 24 h, captured by the QoR-15 nausea and vomiting item, showed no statistically significant between-group difference (median 10 [IQR 6–10] vs. 10 [IQR 5–10]; *p* = 0.672); postoperative nausea and vomiting was not otherwise recorded systematically. The corresponding baseline-adjusted QoR-15 results are summarized in Table 5.

### 3.6. Exploratory QoR-15 Subdomain Analysis

Among the five QoR-15 subdomains, physical comfort (Q1–Q4: breathing, food enjoyment, rest, sleep) showed the largest between-group difference, with a baseline-adjusted difference of 4.53 points favouring pre-incision timing (95% CI 0.63 to 8.43; raw *p* = 0.024). After Benjamini–Hochberg correction across the five subdomains, this difference was no longer significant (adjusted *p* = 0.119). The other four subdomains showed no statistically significant between-group difference (Table 6, Figure 5). These subdomain analyses were exploratory; none was confirmatory, and no item-level comparison was performed.

### 3.7. Correlation Analysis

Exploratory correlations between analgesic outcomes and quality of recovery were weak and non-significant: total 24 h IV-MME versus QoR-15 change (ρ = −0.23; *p* = 0.095) and NRS at PACU admission versus QoR-15 change (ρ = −0.20; *p* = 0.146) (Figure 6). This is consistent with the view that pain scores and opioid consumption capture different aspects of recovery from those measured by a multidimensional patient-reported instrument such as the QoR-15 [27]. The association between PACU NRS and rescue fentanyl is not reported as a study finding, because fentanyl was administered according to an NRS-triggered rescue protocol and this relationship is therefore built into the treatment algorithm.

## 4. Discussion

In this retrospective cohort of 54 patients undergoing laparoscopic sleeve gastrectomy, pre-incision administration of combined external oblique intercostal and rectus sheath blocks was associated with lower pain scores throughout early recovery and with lower recorded opioid consumption over the first 24 h. The association persisted after adjustment for BMI, ASA physical status and anaesthesia duration in a generalized estimating equations model. The magnitude of the difference was greatest at PACU admission and attenuated progressively thereafter. No statistically significant between-group difference in overall quality of recovery was detected with the QoR-15, although the estimate was imprecise; an exploratory subdomain analysis suggested better physical comfort with pre-incision timing, but this did not survive correction for multiple comparisons.

Pain was rated on a 0 to 10 numerical rating scale, and the size of the between-group difference is as informative as its statistical significance. Patients in the pre-incision group scored 2.42 points lower at PACU admission (95% CI −3.72 to −1.12). In postsurgical patients with moderate acute pain, a decline of approximately 1.3 points corresponds to minimal improvement and one of approximately 2.4 points to much improvement [32]. A difference of this magnitude at emergence is therefore compatible with a clinically important effect, although those thresholds were derived from within-patient change rather than from between-group comparison. By 24 h, the difference had fallen to 0.81 points (95% CI −1.30 to −0.33). This remained statistically significant, but it is smaller than the change patients describe as even minimal improvement, and by that time, both groups reported only mild pain (median 1 versus 2 on the 0 to 10 scale). The late difference is therefore unlikely to be clinically meaningful, and the between-group difference of potential clinical importance was concentrated in the early recovery period. This pattern is most simply explained by the block being already established at emergence, rather than by a specifically pre-emptive effect. A block completed before incision has had the whole operation in which to establish its effect and is fully developed when the patient emerges, whereas a block placed at wound closure is administered only minutes before extubation and may still be developing when the patient reaches the PACU. In this cohort, blocks performed at the end of surgery were followed by extubation within a short interval, whereas in the pre-incision group, the block was followed by patient positioning, skin preparation and draping before incision. The exact intervals from block completion to incision, extubation and PACU admission were not documented in the medical record and therefore cannot be reported, which limits formal evaluation of this explanation. It should also be noted that the time to peak plasma concentration after fascial plane block administration reflects systemic absorption from the injection plane and is not a measure of the time to onset or to maximal neural blockade; plasma pharmacokinetics should not be used as a surrogate for analgesic onset. An additional contribution from attenuation of intraoperative nociceptive input, and thereby of central sensitisation, remains biologically plausible, but the present design cannot separate such an effect from the difference in pharmacodynamic onset. We therefore describe the groups by the timing of administration, pre-incision and post-surgery, rather than as pre-emptive and non-pre-emptive.

Our findings complement, rather than duplicate, the existing randomised evidence. Recent randomised controlled trials have examined the EOI block in laparoscopic sleeve gastrectomy against control, against port-site infiltration and against an alternative fascial plane technique [16,17,18,19], and a meta-analysis confirms its opioid-sparing effect [20]. However, none of these trials examined when the block should be administered. Our findings identify block timing as a candidate factor for prospective evaluation. Administering an established block before incision, rather than at closure, was associated with lower early pain scores and reduced opioid requirements. Direct evidence on block timing remains limited, and the two available randomised trials disagree. In a randomised trial of 200 patients undergoing laparoscopic cholecystectomy, Jeong et al. [33] found that a rectus sheath block placed before skin incision significantly reduced total 24 h rescue analgesic consumption compared with the identical block performed after skin closure, a result concordant with our findings. In contrast, Acar et al. [34] randomised patients undergoing laparoscopic cholecystectomy to erector spinae plane block administered either before induction or after surgery and found no significant difference in postoperative pain scores or 24 h tramadol consumption, although preoperative administration was associated with more favourable intraoperative haemodynamics. Several factors may explain this discordance. The erector spinae plane block and the rectus sheath or external oblique intercostal blocks differ in depth, volume of distribution and expected onset, and the surgical insult of laparoscopic cholecystectomy is smaller than that of sleeve gastrectomy; any timing-related difference in pharmacodynamic readiness at emergence would therefore be expected to be smaller and harder to detect. Taken together, the randomised evidence does not establish that earlier administration is universally advantageous, and our observational findings should be read as one contribution to an unresolved question rather than as confirmation of a general principle. Wider reviews of fascial plane block techniques for upper abdominal surgery similarly note that comparative and procedural questions, including timing, remain incompletely answered [35]. To our knowledge, ours is the first study to address block timing specifically in bariatric patients, for whom minimizing opioid exposure carries particular clinical importance [4,5].

A notable discordance was observed between the NRS findings and the QoR-15 pain subdomain (Q11–Q13). Despite large and sustained between-group NRS differences at every PACU time point, the QoR-15 pain items showed no significant separation at 24 h. This is most plausibly explained by the differing temporal frameworks of the two instruments; the NRS captured pain intensity at discrete, early time points (predominantly during the first 60 min after emergence, when the between-group difference was greatest), whereas the QoR-15 pain domain reflects the patient’s global experience across the preceding 24 h. As NRS scores converged to below 3 by 6 h, the pronounced early difference was diluted when integrated over a full day.

The physical comfort subdomain finding is of interest given that it did not coincide with a difference in the QoR-15 pain items. One interpretation is that superior early pain control reduces the physiological burden of the emergence period, with downstream effects on sleep quality and food tolerance in the early postoperative hours, outcomes that align with enhanced recovery targets [6]. The physical comfort domain has previously been identified as responsive to perioperative interventions; Kim et al. [36] observed improvement in the comfort dimension of the QoR-40 with serratus plane blocks, Castro-Alves et al. [37] reported improvement across all QoR-40 dimensions with neuraxial anaesthesia, and Chazapis et al. [38] noted that rest and sleep items in the QoR-15 carry high sensitivity to analgesic interventions. Whether our finding represents a direct consequence of reduced opioid consumption, a pre-emptive effect on central sensitisation [39,40], or both, cannot be determined from this design. It is also informative that no statistically significant between-group difference in overall QoR-15 was detected here, whereas Ibrahim et al. [8] reported improved quality of recovery after sleeve gastrectomy when loco-regional blocks were combined with an opioid-free anaesthetic technique. Their intervention modified the entire anaesthetic strategy, rather than only the timing of an otherwise identical block, which may explain why a difference in global recovery emerged in their randomised trial but not in our timing comparison.

The 35% reduction in total 24-h opioid consumption was driven primarily by the substantially greater rescue fentanyl requirement in the post-surgery group during the PACU period (81 ± 67 vs. 146 ± 56 µg), combined with lower PCA tramadol use in the pre-incision group over the ward period (128 ± 37 vs. 174 ± 45 mg; *p* < 0.001). From a clinical safety perspective, avoiding large-dose intravenous opioid rescue during the immediate postoperative period is particularly relevant in bariatric patients given their elevated risk of opioid-induced respiratory depression related to obstructive sleep apnoea [4,5].

Several limitations should be noted. This was a retrospective cohort study conducted at a single centre, and allocation to timing groups was not randomised. Block timing reflected naturally occurring logistical variation in clinical practice; it depended on the availability of the regional anaesthesia team on the day of surgery rather than on any patient- or case-related clinical criterion. This is not randomisation, and it cannot be assumed to share its properties. Operating-room workload, case sequence, time of day, the complexity of preceding cases, staffing patterns and list compression may themselves correlate with aspects of perioperative management, and none of these was captured in the dataset. Baseline characteristics were comparable between groups on all measured variables, including age, sex, BMI, ASA physical status, documented comorbidity, operative and anaesthesia duration and preoperative QoR-15 scores, and the two timings were evenly distributed across calendar quarters and days of the week. However, non-significant differences in a sample of this size do not establish exchangeability between groups, and measured baseline balance does not prove the absence of selection bias. Residual and unmeasured confounding therefore remains possible, and all findings should be interpreted as associations rather than as causal effects. Potential unmeasured confounders include intraoperative opioid dose adjustments, patient anxiety level, baseline pain sensitivity, duration of preoperative fasting, and surgeon-related technical factors such as port placement and trocar size. Ward nursing staff conducting postoperative assessments were not informed of block timing, providing operational blinding for outcome assessment; however, this does not constitute prospective study-level blinding. All blocks were performed by two experienced anaesthesiologists throughout the study period, reducing operator variability within this cohort.

The female proportion in our cohort (55.6%) was lower than that reported in contemporary bariatric populations [41]. Consecutive eligible patients were included without matching, and this difference may reflect the referral pattern of our setting, although the reason cannot be established from the present data. As pain perception and opioid requirements may differ by sex, this may limit the generalizability of our findings to predominantly female bariatric cohorts. Sex distribution was similar between the timing groups (*p* = 1.00); nevertheless, residual confounding by sex cannot be excluded.

Local anaesthetic safety requires explicit comment. The combined bupivacaine dose exceeded the commonly cited maximum recommended dose of 2 mg/kg in some lighter patients once trocar-site infiltration was included (overall mean 2.28 mg/kg; maximum 3.65 mg/kg). Although these thresholds derive largely from single-injection scenarios, this does not establish that the present regimen was safe. Recent pharmacokinetic work on fascial plane blocks emphasises that high-volume injections into vascular planes can produce unexpectedly high systemic concentrations in individual patients, and recommends weight-adjusted dosing, incremental administration, close monitoring and keeping total exposure within accepted limits [42,43]. Absorption from multiple compartments can overlap during prolonged uptake from fascial planes; so, the fact that the two components were given at different sites and times does not by itself guarantee a lower peak concentration. This study included no systematic surveillance for local anaesthetic systemic toxicity, no plasma bupivacaine measurements, no prospective neurological assessment and no predefined safety endpoint. Accordingly, the only defensible statement is that no clinically apparent event suggestive of local anaesthetic systemic toxicity was identified on retrospective review of the anaesthetic and recovery records; safety was not demonstrated. Units performing high-volume fascial plane blocks in bariatric patients should apply weight-based dose limits and maintain readiness to manage systemic toxicity in accordance with current guidance [44]. The absence of a prospective safety protocol is a limitation, and future studies should incorporate systematic surveillance for local anaesthetic systemic toxicity.

Our multimodal protocol included a single perioperative dose of intravenous ibuprofen (800 mg). The routine use of NSAIDs after bariatric surgery is debated, although the association with marginal ulceration derives predominantly from Roux-en-Y gastric bypass, and a large registry-based cohort found no comparable increase in peptic ulcer risk after sleeve gastrectomy [45]. Because the identical NSAID protocol was applied to both timing groups, it cannot account for the between-group differences reported here.

A specific and important limitation is that intraoperative opioid exposure could not be quantified. All patients received fentanyl at induction and remifentanil titrated to surgical stimulation, and no other intraoperative opioid was given, but cumulative remifentanil dose was not recorded in a retrievable form. This matters in two ways; a functioning pre-incision block might reduce intraoperative remifentanil requirements, placing intraoperative opioid exposure on the causal pathway as a mediator rather than a simple confounder; and differences in remifentanil exposure could themselves influence early postoperative analgesic requirements through acute tolerance or hyperalgesia. Although institutional practice constrains remifentanil dosing within a customary range, we have no record-level data to support this, and we therefore cannot exclude either mechanism. The intervals from block completion to incision, to extubation and to PACU admission were likewise not documented, which limits formal assessment of the pharmacodynamic-readiness explanation. Several other outcomes recommended for analgesia studies, including oxygen desaturation, respiratory depression, sedation scores, systematically recorded postoperative nausea and vomiting, PACU length of stay, time to ambulation and total hospital stay, were not documented in the source records and could not be analysed. The QoR-15 partially captures functional recovery through its physical independence and physical comfort domains, and its nausea item showed no between-group difference, but dedicated prospective measurement of these safety and recovery endpoints is an important direction for future work. It follows that the reduction in recorded opioid consumption should not be read as evidence of improved respiratory safety or enhanced recovery; opioid exposure was lower, but no opioid-related complication was measured. Similarly, although the rationale for opioid minimisation in bariatric surgery rests partly on the high prevalence of obstructive sleep apnoea, in this cohort, obstructive sleep apnoea was recorded only where previously diagnosed and no systematic screening was undertaken; the documented prevalence of about 5% is therefore far below that expected with formal screening, and statements about the respiratory vulnerability of this particular cohort should be read as general rather than cohort-specific.

Finally, the quality-of-recovery comparison was imprecise. The baseline-adjusted between-group difference of 5.3 points had a 95% confidence interval of −7.0 to 17.6, which spans zero and extends beyond the published minimal clinically important difference in both directions; this study can therefore neither demonstrate nor exclude a clinically relevant effect on overall recovery. For planning purposes only, and assuming the observed standard deviation of 22.7 points, a future trial would require approximately 227 patients per group to detect a 6-point difference, or 128 per group to detect an 8-point difference, with 80% power at a two-sided alpha of 0.05. The physical comfort subdomain result did not survive correction for multiple comparisons and should be regarded as a signal to be tested prospectively, not as a finding. QoR-15 was measured only at 24 h; adding a 48 h measurement would provide a more complete recovery picture [38]. The QoR-15 was administered using the validated Turkish version (QoR-15T) [29,30], which has demonstrated acceptable psychometric properties in Turkish-speaking surgical patients.

## 5. Conclusions

In this retrospective single-centre cohort of patients undergoing laparoscopic sleeve gastrectomy, administration of combined external oblique intercostal and rectus sheath blocks before incision was associated with lower pain scores during early postoperative recovery and lower recorded opioid consumption over the first 24 h compared with block administration at the end of surgery. The difference was largest at emergence and attenuated over the first postoperative day. No statistically significant difference was observed in overall QoR-15 recovery scores, although the confidence interval was wide and did not exclude a clinically relevant difference. Because treatment timing was not randomised and because potentially important perioperative confounders, in particular, intraoperative opioid exposure, could not be accounted for, these findings identify a question for prospective testing rather than establishing an effect of timing. Prospective randomised trials incorporating standardised local anaesthetic dosing, measurement of intraoperative opioid exposure, clinically relevant recovery and respiratory outcomes, and predefined safety surveillance are required to determine whether block timing independently improves postoperative recovery.

## Figures and Tables

**Table 1:** Patient demographics and baseline characteristics.

Variable	Pre-Incision (*n* = 28)	Post-Surgery (*n* = 26)	*p*-Value
Age (years)	39.5 ± 13.3	36.7 ± 13.9	0.467
Sex, male/female	12/16	12/14	1.000
Height (cm)	167.8 ± 9.4	171.0 ± 9.8	0.260
Weight (kg)	118.3 ± 21.3	120.0 ± 24.8	0.993
BMI (kg/m^2^)	42.0 ± 6.7	40.7 ± 5.6	0.782
ASA, II/III	20/8	19/7	0.766
Anaesthesia duration (min)	214 ± 35	202 ± 31	0.371
Surgery duration (min)	163 ± 38	171 ± 37	0.456
Baseline QoR-15	124.4 ± 18.7	123.2 ± 17.7	0.842
Hypertension, *n* (%)	12 (42.9)	9 (34.6)	0.586
Diabetes mellitus, *n* (%)	7 (25.0)	7 (26.9)	1.000
Obstructive sleep apnoea, *n* (%) †	2 (7.1)	1 (3.8)	1.000
Any documented comorbidity, *n* (%)	18 (64.3)	14 (53.8)	0.580

Data are mean ± SD or *n* (%). All patients underwent laparoscopic sleeve gastrectomy. Categorical comparisons by Fisher’s exact test. † Recorded where previously diagnosed; no systematic screening was performed, so this figure underestimates true prevalence.

**Table 2:** NRS pain scores at rest and opioid consumption.

Variable	Pre-Incision (*n* = 28)	Post-Surgery (*n* = 26)	*p*-Value
NRS at rest, median (IQR)			
PACU 0 min *	4.5 (2.0–8.0)	7.0 (7.0–8.0)	0.008
PACU 15 min	4.5 (2.0–6.3)	7.0 (6.0–7.0)	0.001
PACU 30 min	2.0 (2.0–5.0)	6.0 (4.0–7.0)	<0.001
PACU 45 min	2.0 (1.8–4.0)	5.0 (3.0–6.0)	0.010
PACU 60 min	2.0 (1.0–3.0)	3.0 (2.0–5.0)	0.011
6 h	2.0 (1.0–2.0)	3.0 (2.0–3.0)	0.003
24 h	1.0 (0.0–2.0)	2.0 (1.0–2.0)	0.002
Opioid consumption, median (IQR)			
PACU fentanyl (µg)	62.5 (25.0–106.3)	150.0 (100.0–193.8)	<0.001
PCA tramadol, 24 h (mg)	120.0 (100.0–150.0)	172.5 (141.3–198.8)	<0.001
Total 24 h IV-MME (mg)	19.5 (15.8–27.0)	33.5 (25.9–37.0)	<0.001

* Primary outcome. IV-MME, intravenous morphine milligram equivalents. All *p*-values from Mann–Whitney U tests, reported for description; inference for the repeated pain measurements is based on the models in Table 3 and Table 4. Corresponding means ± SD are given in the text.

**Table 3:** Generalized estimating equations model for repeated NRS pain scores.

Parameter	Coefficient	95% CI	*p*-Value
Model A—Unadjusted			
Pre-incision timing	−1.59	−2.23 to −0.95	<0.001
Time: PACU 15 min vs. 0 min	−0.54	−1.07 to −0.00	0.049
Time: PACU 30 min vs. 0 min	−1.80	−2.53 to −1.07	<0.001
Time: PACU 45 min vs. 0 min	−2.43	−3.18 to −1.67	<0.001
Time: PACU 60 min vs. 0 min	−3.24	−3.95 to −2.53	<0.001
Time: 6 h vs. 0 min	−3.94	−4.68 to −3.21	<0.001
Time: 24 h vs. 0 min	−4.63	−5.36 to −3.90	<0.001
Model B—Adjusted			
Pre-incision timing	−1.53	−2.15 to −0.91	<0.001
Time: PACU 15 min vs. 0 min	−0.54	−1.07 to −0.00	0.049
Time: PACU 30 min vs. 0 min	−1.80	−2.53 to −1.07	<0.001
Time: PACU 45 min vs. 0 min	−2.43	−3.18 to −1.67	<0.001
Time: PACU 60 min vs. 0 min	−3.24	−3.95 to −2.53	<0.001
Time: 6 h vs. 0 min	−3.94	−4.68 to −3.21	<0.001
Time: 24 h vs. 0 min	−4.63	−5.36 to −3.90	<0.001
BMI (per kg/m^2^)	+0.032	−0.014 to +0.077	0.171
ASA III vs. II	−0.004	−0.742 to +0.734	0.992
Anaesthesia duration (per min)	−0.008	−0.017 to +0.000	0.058

Generalized estimating equations, Gaussian distribution with identity link, time entered as a categorical factor (PACU admission as reference), exchangeable working correlation structure (estimated within-patient correlation 0.32) and robust standard errors. Model B is additionally adjusted for BMI, ASA physical status and anaesthesia duration. A negative coefficient indicates a lower NRS score. Covariates were included for confounding adjustment and are not interpreted as independent predictors.

**Table 4:** Model-based between-group difference in NRS at each time point.

Time Point	Difference	95% CI	*p*-Value
PACU 0 min	−2.42	−3.72 to −1.12	<0.001
PACU 15 min	−2.19	−3.22 to −1.16	<0.001
PACU 30 min	−2.11	−3.15 to −1.06	<0.001
PACU 45 min	−1.53	−2.63 to −0.44	0.006
PACU 60 min	−1.25	−2.22 to −0.28	0.012
6 h	−0.83	−1.34 to −0.31	0.002
24 h	−0.81	−1.30 to −0.33	<0.001

Estimates derived from a generalized estimating equations model including a group  ×  time interaction. A negative value indicates a lower NRS score in the pre-incision group. Group  ×  time interaction: Wald χ^2^ = 11.47, df = 6, *p* = 0.075.

**Table 5:** QoR-15 scores and baseline-adjusted between-group differences.

QoR-15 Score	Pre-Incision	Post-Surgery	Adjusted Difference	95% CI	*p*
Baseline (preoperative)	124.4 ± 18.7	123.2 ± 17.7	—	—	0.842
24 h	113.5 ± 29.4	107.3 ± 21.9	5.3	−7.0 to 17.6	0.391
Part A (well-being)	—	—	4.5	−3.8 to 12.9	0.282
Part B (symptoms)	—	—	1.0	−5.8 to 7.7	0.778

Scores are mean ± SD. Adjusted difference is the between-group difference in the 24-h score from an analysis of covariance with the corresponding baseline score as covariate; a positive value favours pre-incision timing. Baseline scores were compared with the Mann–Whitney U test. QoR-15 range 0–150.

**Table 6:** Baseline-adjusted between-group differences in QoR-15 subdomain scores (exploratory analysis).

Subdomain	Adjusted Difference	95% CI	Raw *p*	Adj. *p*
Physical comfort	+4.53	+0.63 to +8.43	0.024	0.119
Emotional state	+0.27	−2.55 to +3.10	0.846	0.846
Physical independence	−1.08	−4.02 to +1.87	0.467	0.805
Psychological support	+0.40	−1.02 to +1.81	0.577	0.805
Pain	+0.76	−2.51 to +4.02	0.644	0.805

Between-group differences in the 24 h subdomain score, adjusted for the corresponding baseline subdomain score by analysis of covariance. A positive value favours pre-incision timing. Adj. *p*, Benjamini–Hochberg adjusted *p*-value across the five subdomains. These analyses are exploratory.

## Data Availability

The data presented in this study are available on request from the corresponding author. The data are not publicly available due to institutional patient privacy restrictions.
